# An Uncommon Case of Dulaglutide-Related Morbilliform Drug Eruption

**DOI:** 10.7759/cureus.21536

**Published:** 2022-01-23

**Authors:** Georgios Kyriakos, Evangelos Diamantis, Eleni Memi, Ioannis Elefsiniotis

**Affiliations:** 1 Endocrinology and Nutrition Section, Hospital General Universitario Santa Lucía, Cartagena, ESP; 2 Academic Department of Internal Medicine - Endocrinology Unit, General Oncology Hospital of Kifisia "Agioi Anargyroi" National and Kapodistrian University of Athens, Athens, GRC; 3 Unit of Endocrinology, Diabetes Mellitus and Metabolism, Aretaieion University Hospital, Medical School of Athens, National and Kapodistrian University of Athens, Athens, GRC; 4 Academic Department of Internal Medicine, General Oncology Hospital of Kifisia "Agioi Anargyroi" National and Kapodistrian University of Athens, Athens, GRC

**Keywords:** types 2 diabetes, cutaneous adverse drug reaction, glucagon-like peptide-1 receptor agonist, morbilliform drug eruption, skin rash, dulaglutide

## Abstract

Dulaglutide is a once-weekly injectable glucagon-like peptide-1 (GLP-1) receptor agonist that has shown a durable glycemic efficacy as well as beneficial effects on body weight and major adverse cardiovascular events (MACE) outcomes, making it an important option for the treatment of type 2 diabetes. Common side effects of dulaglutide include nausea, diarrhea, and abdominal distension, and these are usually mild to moderate in severity and tend to diminish over time. Morbilliform drug eruptions to dulaglutide are very rare, with only one case reported until now. We report another case of dulaglutide-morbilliform drug eruption to alert the attending physicians that dulaglutide-related adverse skin reactions should be kept in mind as generalized use of dulaglutide and other GLP-1 receptor agonists are expected to remain in widespread clinical use in the future.

## Introduction

Glucagon-like peptide-1 (GLP-1) receptor agonists are among the most effective glucose-lowering medications and weight-loss drugs indicated for the treatment of type 2 diabetes [1]. Dulaglutide is a once-weekly injectable GLP-1 medication that has been shown to reduce hemoglobin A1c (HbA1c), body weight, and blood glucose levels among patients with type 2 diabetes [2]. Furthermore, in the Researching Cardiovascular Events With a Weekly Incretin in Diabetes (REWIND) trial, dulaglutide was shown to reduce the first occurrence of the 3-point major adverse cardiovascular events (MACE) composite outcome including stroke, and improve kidney function during a median follow-up of 5.4 years [3]. Side effects of dulaglutide are mainly gastrointestinal in nature, such as nausea and abdominal discomfort, and generalized cutaneous adverse reactions are extremely uncommon [4]. In this report, we present a rare case of a dulaglutide-related morbilliform drug eruption.

## Case presentation

**Figure 1:**
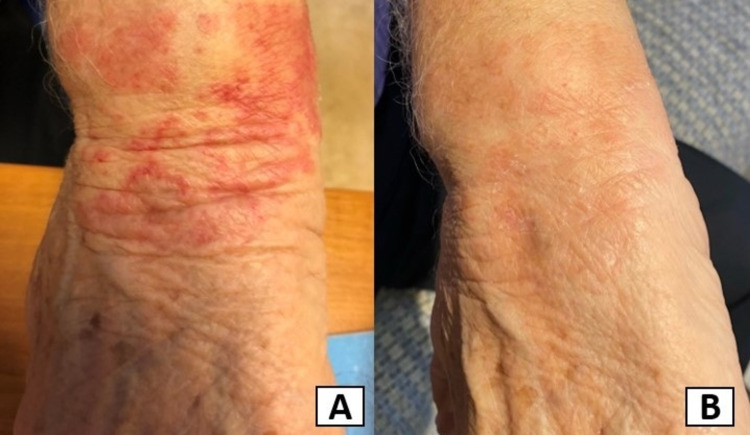
A. Red macules and papules involving the right upper extremity. B. The skin rash quickly improved following the cessation of dulaglutide and the treatments indicated

A 75-year-old overweight male patient with a medical history of type 2 diabetes mellitus, hyperuricemia, and hypertension was referred to the outpatient clinic of the Endocrinology Unit for improvement in glycemic control. The patient was receiving glargine as basal insulin 20 units per day at bedtime, metformin 1 g twice per day, and repaglinide 1 mg before breakfast, lunch, and dinner. The most relevant lab tests were as follows: HbA1c: 8.3%, glomerular filtration rate (GFR): 50, C-peptide: 2.1 ng/mL, low-density lipoprotein (LDL): 71 mg/dl. The patient reported high post-prandial glucose levels, normally above 200 mg/dl. Repaglinide was withdrawn and dulaglutide was initiated at a dose of 1.5 mg subcutaneously once a week. A month later at the follow-up appointment, the patient reported improved glycemic control as well as the appearance of a rash on his arms and legs that had started two weeks after the initiation of dulaglutide (Figure 1A).

He reported no recent illnesses or tick bites. The patient reported no arthralgias, myalgias, pruritus, or gastrointestinal symptoms. The main findings of the clinical examination were macules and papules that affected the patient's upper and lower extremities. The patient was advised to apply betamethasone valerate cream 0.1% twice a day to the affected areas and was instructed to discontinue dulaglutide. Two weeks after the cessation of dulaglutide, the skin rash had almost disappeared (Figure 1B). The patient refused a rechallenge.

## Discussion

Dulaglutide is considered an important option in the management of type 2 diabetes given its durable glycemic efficacy and beneficial effects on body weight and MACE outcomes, the low inherent risk of hypoglycemia, and convenient once-weekly regimen [1]. The most common side effects of dulaglutide include gastrointestinal disorders like nausea, vomiting, and diarrhea. However, these effects appear to be mostly mild-to-moderate in severity, and they appear to lessen over time. The chief cutaneous side effects are injection site reactions and are typically mild, itchy, erythematous, and transient [2].

Naranjo Scale was developed to help standardize the assessment of causality for all adverse drug reactions [5]. Probability is assigned via a score based on a questionnaire that assesses the temporal sequence of medication with the adverse event, resolution of symptoms with medication discontinuation, and no alternative diagnosis possible based on medical history, clinical examination, and diagnostic workup. In our patient, dulaglutide was assumed to be the cause of the morbilliform eruption based on the Naranjo Scale.

Morbilliform drug eruptions to dulaglutide are very rare with only one case described in the literature until now [6]. Rzepka et al. have reported a generalized cutaneous drug reaction to dulaglutide in an 84-year-old patient with the appearance of macules and papules in a morbilliform pattern in the patient's lower extremities, abdomen, neck, upper extremities, and back with purpuric discoloration of dependent lesions on his lower extremities and non-blanching petechiae of his bilateral palms [6].

The mechanism of dulaglutide-induced morbilliform drug eruption is unclear. GLP-1 receptor is expressed in murine skin around hair follicles [7]. Evidence also suggests limited GLP-1 receptor expression in human keratinocyte and skin fibroblasts, which increases with active inflammation [8]. GLP-1 agonists may decrease dermal γδ T-cell number and IL-17 expression in psoriasis plaques, and various reports have described long-lasting improvement of plaque psoriasis with the administration of GLP-1 agonists in patients with type 2 diabetes [9,10]. However, cases of skin disorders such as bullous pemphigoid have been reported in elderly patients with type 2 diabetes after treatment with dulaglutide and other GLP-1-receptor agonists [11]. Interestingly, GLP-1 expression has also been associated with pyoderma gangrenosum, and cases of vesiculopustular dermatosis have been reported with the use of GLP-1 agonists [12,13].

## Conclusions

This case is of interest as it presents a rare dulaglutide-related adverse skin reaction, which should be kept in mind by the attending physicians as dulaglutide and other GLP-1 agonists are expected to remain in widespread clinical use in the future. This will help to recognize it and treat it as soon as possible. Further study of GLP-1 agonist and GLP-1 receptor expression in cutaneous tissue is warranted in order to determine the mechanism of the development of these skin lesions.
